# CyLineUp: A Cytoscape app for visualizing data in network small multiples

**DOI:** 10.12688/f1000research.8402.1

**Published:** 2016-04-11

**Authors:** Maria Cecília D. Costa, Thijs Slijkhuis, Wilco Ligterink, Henk W.M. Hilhorst, Dick de Ridder, Harm Nijveen

**Affiliations:** 1 Wageningen Seed Lab, Laboratory of Plant Physiology, Wageningen University, Wageningen, Netherlands; 2 Bioinformatics Group, Wageningen University, Wageningen, Netherlands

**Keywords:** Data display, Expression profiling, Graphical user interfaces, Metabolite profiling, Small multiples

## Abstract

CyLineUp is a Cytoscape 3 app for the projection of high-throughput measurement data from multiple experiments/samples on a network or pathway map using “small multiples”. This visualization method allows for easy comparison of different experiments in the context of the network or pathway. The user can import various kinds of measurement data and select any appropriate Cytoscape network or WikiPathways pathway map. CyLineUp creates small multiples by replicating the loaded network as many times as there are experiments/samples (e.g. time points, stress conditions, tissues, etc.). The measurement data for each experiment are then mapped onto the nodes (genes, proteins etc.) of the corresponding network using a color gradient. Each step of creating the visualization can be customized to the user’s needs. The results can be exported as a high quality vector image.

## Introduction

Developments in high-throughput -omics measurement techniques have allowed researchers to routinely obtain presence/absence, levels or interactions of molecules at a genome-wide scale, often addressing important questions that could not be answered before. Consequently, the demand for more and better tools for data analysis is growing, especially visualization tools to project data of multiple samples (e.g. different time points or tissues) on network or pathway maps. Visualization plays an important role in data analysis as a way to obtain insights, select details and present findings ^1^.

Several software tools enable visualization of expression data on pathways ^2– 4^ and the functionality to plot charts on nodes has become a standard feature of Cytoscape 3 ^5^. While these visualizations often work well for analyzing measurement patterns of individual genes in a network, they do not easily show changes at a more global level in the network. Often, these global effects are the most interesting to the biologist.

Here, we present a different approach that uses “small multiples”. This approach is a visualization technique that shows multiple copies of a graph with the same combination of variables, but with different changes in each variable ^6^. Small multiples facilitate comparison, presentation, storytelling and search for patterns, trends and outliers ^1^.

There is currently no visualization tool enabling projection of data from different experiments/samples on small multiples. Therefore, we developed the Cytoscape app CyLineUp, that takes advantage of the tools for importing and displaying maps integrated into Cytoscape to create small multiples. We applied CyLineUp to visualize changes in the glycolysis/tricarboxylic acid (TCA) cycle induced by the phytohormone abscisic acid (ABA) in germinated *Arabidopsis thaliana* seeds.

## Methods and implementation

### Implementation

CyLineUp was written in Java 7 as an OSGi (Open Services Gateway Initiative) bundle. It adds a “CyLineUp” tab to the Cytoscape “Control Panel” offering functionality to import the data, configure the views and visual styles, and export an SVG rendering of the visualization. The data import functionality is implemented as a Cytoscape Task, using Tunable annotations for user input dialogs. The small multiples visualization is implemented as a set of views on the same underlying network. Each view has its own visual style for coloring the nodes according to the input data. The FreeHEP Graphics 2D Library is used for exporting an SVG image with the small multiples.

### Operation

**Figure 1.**
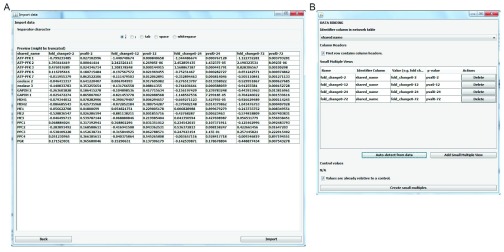
Importing data. ( **A**) After selecting the text file with the input data, the user should select the field/column separator character (comma, semicolon, tab or whitespace). ( **B**) On the data binding panel, the user should select the column that contains the identifier (“shared name” in our example) and configure the small multiples either by using the auto-detect functionality or manually (add small multiple views).

**Figure 2.**
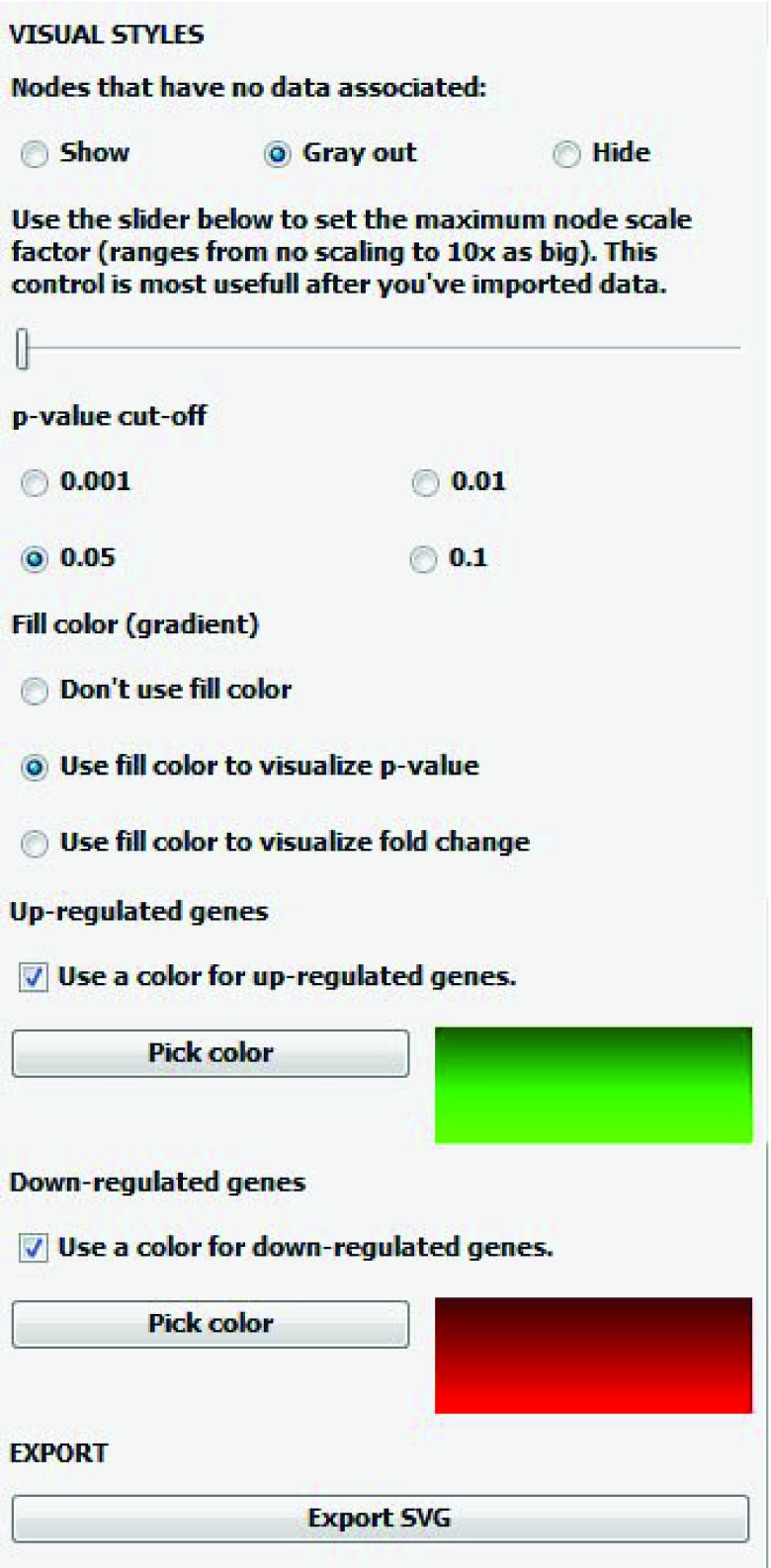
Configuring the visual styles. The user can customize several visualization settings by changing the Visual styles. Nodes that have no data associated can be shown (default), greyed out or hidden. For hidden nodes, the associated edges will also be hidden. The node color can be set to reflect the p-value cut-off (0.001, 0.01, 0.05 and 0.1) or fold change. The default colors are green (HEX #00FF00) for “up-regulated genes” and red (HEX #FF0000) for “down-regulated genes”. The user can choose different colors by pressing the Pick color buttons. After customization, the user can export the visualization to an SVG image file.

Visualizing -omics data with CyLineUp consists of four main steps. (I) Creating the map or network that will be the basis for data visualization. A large collection of pathways from the WikiPathways platform ( http://wikipathways.org) can be easily imported using the WikiPathways Cytoscape app ^7^. Optionally, pathways created and imported directly from Pathvisio ^8^ and networks created in Cytoscape can be used. The users should take care that the names of the identifiers are the same in the data file and in the pathway/network. (II) Importing data and linking it to the map using the graphical user interface provided by CyLineUp ( Figure 1). CyLineUp accepts text files with comma, semicolon, tab or arbitrary whitespaces separated values. CyLineUp provides auto-import functionality, but manual selection of data is also possible. The auto-import functionality is based on the order of the columns in the data file. Therefore, it is advisable to have the identifiers in the first column and the values (e.g. fold change) and the p-values for each treatment/sample in subsequent columns. (III) Customizing the visualization to the user’s needs by changing the Visual styles in the CyLineUp user interface ( Figure 2). Nodes that have no data associated can be shown (default), greyed out or hidden. In case the nodes are hidden, the edges associated to them will also be hidden. The default colors are green (HEX #00FF00) for up-regulated genes and red (HEX #FF0000) for down-regulated genes. The user can chose different colors by pressing the *Pick color* buttons. After each change, the user can update the view of the small multiples by pressing the button *Update network views*. (IV) Exporting the visualization to an SVG image file. A real-time image preview is available, but can be disabled in case it takes too much computer resources.

**Figure 3.**
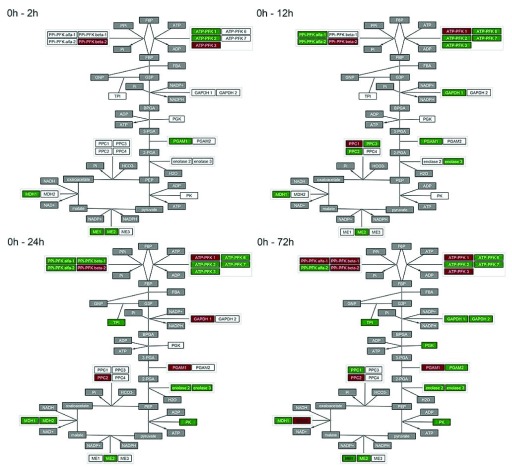
Small multiples showing temporal changes in the expression of genes acting in the glycolysis/TCA cycle induced in germinated *A. thaliana* seeds by incubation in ABA for 0, 2, 12, 24 and 72 h. A general activation of the cycle can be observed and may be a mechanism by which the plant cell prepares for the energy demands of recovery from drought. Red nodes indicate genes with significantly declining transcript abundance. Green nodes indicate genes with significantly accumulating transcripts. White nodes indicate genes without significant changes in transcript abundance. Grey nodes indicate components that are not genes or do not have data associated.

### Use case

We used available gene expression data from a time series of microarrays ^9^ to validate the use of CyLineUp. The data were generated to explore the temporal changes induced by ABA in germinated *A. thaliana* seeds ^9^. Seeds at the stage of radicle protrusion (0 h) and after four periods (2, 12, 24 and 72 h) of incubation in ABA were used. Details about data acquisition and processing can be found in Costa *et al.* ^9^. Here, we used CyLineUp to explore the main changes in the glycolysis/TCA cycle during ABA incubation ( Figure 3).

The map of the cycle was created using Pathvisio 3.2.0 ^8^ and imported into Cytoscape 3.2.1 ( Supplementary file S1.gpml). Then, the expression data and Bonferroni-corrected p-values (comma-separated values (CSV) file: Supplementary file S2.csv) were imported using the CyLineUp user interface. The visual style was set to “grey out” nodes that have no data associated, use a “p-value cut-off” of 0.1 and “use fill color to visualize p-value”.

The glycolysis/TCA cycle provides most of the energy for processes in the seed. A general activation of the cycle could be observed as one of the immediate cellular responses induced by ABA ^10^.

## Conclusion

CyLineUp allows easy visual analysis of data from multiple samples on a biological network to study changes at the (sub-)network level. It is a useful and timely addition to the Cytoscape network analysis platform.

## Software availability

Software available from: http://apps.cytoscape.org/apps/cylineup

Latest source code: https://github.com/Slijkhuis/CyLineUp

Archived source code as at time of publication: http://dx.doi.org/10.5281/zenodo.48135 ^11^

License: Lesser GNU Public License 2.1

## References

[1] van den Elzen S van Wijk JJ: Small multiples, large singles: a new approach for visual data exploration. *Comput Graph Forum.* 2013;32(3pt2):191–200. 10.1111/cgf.12106

[2] Westenberg MA Roerdink JB Kuipers OP: SpotXplore: a Cytoscape plugin for visual exploration of hotspot expression in gene regulatory networks. *Bioinformatics.* 2010;26(22):2922–3. 10.1093/bioinformatics/btq535 20861033 PMC2971575

[3] Yamada T Letunic I Okuda S: iPath2.0: interactive pathway explorer. * Nucleic Acids Res.* 2011;39(Web Server issue):W412–5. 10.1093/nar/gkr313 21546551 PMC3125749

[4] Thimm O Bläsing O Gibon Y: MAPMAN: a user-driven tool to display genomics data sets onto diagrams of metabolic pathways and other biological processes. *Plant J.* 2004;37(6):914–39. [cited 2011 Aug 4]. 10.1111/j.1365-313X.2004.02016.x 14996223

[5] Shannon P Markiel A Ozier O: Cytoscape: a software environment for integrated models of biomolecular interaction networks. *Genome Res.* 2003;13(11):2498–504. 10.1101/gr.1239303 14597658 PMC403769

[6] Tufte ER: The visual display of quantitative information. In: Tufte ER, editor. *The visual display of quantitative information* 2nd ed. Cheshre, Connecticut, Connecticut: Graphics Press,2001;197 Reference Source

[7] Kutmon M Riutta A Nunes N: WikiPathways: capturing the full diversity of pathway knowledge. *Nucleic Acids Res.* 2016;44(D1):D488–94. 10.1093/nar/gkv1024 26481357 PMC4702772

[8] Kutmon M van Iersel MP Bohler A: PathVisio 3: an extendable pathway analysis toolbox. *PLOS Comput Biol.* 2015;11(2):e1004085. 10.1371/journal.pcbi.1004085 25706687 PMC4338111

[9] Costa MC Nijveen H Ligterink W: Time-series analysis of the transcriptome of the re-establishment of desiccation tolerance by ABA in germinated *Arabidopsis thaliana* seeds. *Genom Data.* 2015;5:154–6. Elsevier B.V. 10.1016/j.gdata.2015.06.003 26484244 PMC4583984

[10] Toldi O Tuba Z Scott P: Vegetative desiccation tolerance: Is it a goldmine for bioengineering crops? *Plant Sci.* 2009;176(2):187–99. [cited 2011 Aug 7]. 10.1016/j.plantsci.2008.10.002

[11] Costa MC Slijkhuis T Nijveen H: CyLineUp. *Zenodo.* 2016 Data Source 10.12688/f1000research.8402.1 PMC4909101 27347378

