# Supplementary material for: CyLineUp: A Cytoscape app for visualizing data in network small multiples
Source: F1000Res. 2016 Apr 11;5:635. [Version 1] doi: 10.12688/f1000research.8402.1 (PMC4909101; doi:10.12688/f1000research.8402.1)
Supplement: Peer review history

# Peer review: Referee response for version 1

**DOI:** 10.5256/f1000research.9038.r14169

**Published:** 2016-06-14

**Authors:** Oren Tzfadia

**Affiliations:** 1 Department of Plant Biotechnology and Bioinformatics, Ghent University, Ghent, Belgium

CyLineUp is a new Cytoscape app which allow users to visualize 'changing' networks by enabling  users to match dynamic features to networks.

 In my humble opinion, I find the title a bit confusing - and would modify it to something like:

 "CyLineUp: A Cytoscape app for vizualization of data-matched style in multiple networks".    

 The abstract does a fair job in describing the main features of the app presented in the article. The article include suffice explanations, and usage examples, of the app with real biological data. The app itself is very easy to install and use - and thus is very user friendly, which is always an advantage as Cytoscape is heavily used by no-computer-expert biologists. From my own experience, by following the screenshots and explanations provided in the article, it is easy to plot your own data with CyLineUp. 

 I see the CyLineUp app as a potentially great app for analyzing multiple 'omics' data sets, as often we wish to plot small multiples based on different omics data sets.

I have read this submission. I believe that I have an appropriate level of expertise to confirm that it is of an acceptable scientific standard.

# Peer review: Referee response for version 1

**DOI:** 10.5256/f1000research.9038.r13301

**Published:** 2016-04-19

**Authors:** Alexander Pico

**Affiliations:** 1 Gladstone Institutes, San Francisco, CA, USA

It is a common use case for Cytoscape, to have data for multiple conditions or time points. There are a few other apps to address this in various ways, including cyAnimator, enhancedGraphics and DynNetwork, which utilize either animation or custom chart graphics for nodes. The "small multiples" approach has always been an option for users who were sufficiently skilled with using Cytoscape, which allows one to make multiple networks and apply a unique, data-mapped style to each. This is a tedious and error-prone task, however. So, the CyLineUp app is a welcome tool to make this task easy, consistent and reliable.

The app is easy to install and use, and the paper is clear and well-written. It is nice to see a fundamental visualization technique like "small multiples" applied here. However, it is disappointing to see the red-green color gradient offered by default, which should be shunned by data visualization tool developers. Perhaps something to consider changing in the next release of the app? Since the app is open source, maybe I'll contribute this code patch myself as a pull request!

The CyLineUp app offers its own table importer, visual style editor and image exporter customized for visualizing data for multiple conditions or time points. Looking forward, it would be interesting to see what would be involved in adapting the core of Cytoscape to be able to annotate columns of table data already imported and then support the small multiple view generator, leveraging the native importer, style editor and image exporters. I could picture this functionality becoming a part of the Cytoscape distribution, in the same way the enhancedGraphics app was eventually merged with the core as an out-of-the-box feature. This would make the useful facility of small multiple views available to all users, without having to learn these new, single-purpose interfaces.

I have read this submission. I believe that I have an appropriate level of expertise to confirm that it is of an acceptable scientific standard.
